# Intracardiac "Ghost" Following Implantable Cardioverter-Defibrillator (ICD) Explantation: The Role of Multimodality Imaging in Diagnosis and Management

**DOI:** 10.7759/cureus.109682

**Published:** 2026-05-26

**Authors:** Constantinos Antoniades, Marios Ioannides, Theodoros Christophides, Thrasos Constantinides, Nikolaos Kadoglou, Christos Eftychiou

**Affiliations:** 1 Cardiology Department, Nicosia General Hospital, Nicosia, CYP; 2 Cardiac Magnetic Resonance Imaging Department, Nicosia Heart Center, Nicosia, CYP; 3 Cardiology, University of Cyprus, Nicosia, CYP

**Keywords:** cardiac device infection, cardiac electrophysiology, ghost, icd extraction, infective endocarditis, lead explantation

## Abstract

Intracardiac “ghosts” are mobile remnants that may persist after transvenous lead extraction (TLE) and can mimic vegetations on transesophageal echocardiography. We report a 55-year-old man who underwent implantable cardioverter-defibrillator (ICD) explantation for methicillin-resistant Staphylococcus aureus (MRSA) bacteremia. Post-explantation echocardiography revealed a large mobile mass attached to the tricuspid annulus and right atrial appendage. Cardiac magnetic resonance (CMR) imaging demonstrated a nonperfused structure consistent with an organized fibrotic sheath. Serial imaging showed spontaneous regression, although a small pulmonary embolism occurred. This case highlights the echocardiographic features and clinical evolution of a post-explantation “ghost.”

## Introduction

Transvenous lead extraction (TLE) is a well-established strategy for treating cardiac implantable electronic device (CIED) infections, lead malfunction, venous occlusion, and various complications related to leads [1-4]. The growing number of pacemakers and implantable cardioverter-defibrillators (ICDs) used globally corresponds to an increasing incidence of lead extraction procedures or revisions [1,2]. To distinguish between extraction and explantation, lead extraction indicates the complex removal of leads implanted for over one year, whereas lead explantation suggests the removal of leads implanted less than one year ago using simple techniques with standard tools [1].

After lead extraction/explantation, residual intracardiac masses, or “ghosts,” can sometimes be observed during echocardiography. According to the literature, ghosts are formed secondary to a long-standing mechanical interaction between a lead and the endocardium, which causes endocardial injury, fibrin deposition, thrombus organization, inflammation, and finally progressive formation of a tubular fibrotic capsule that is left attached to intracardiac structures after lead removal [5-7]. In other words, the fibrotic mass remains in the vicinity of the lead site after extraction while maintaining its tubular shape [3,5,6].

The estimated prevalence of ghosts ranges approximately from 8% to 14% when systematic postprocedural echocardiographic control is routinely performed [4,6,7]. Generally speaking, ghosts appear to be mostly harmless findings; however, several research studies have found a correlation with increased mortality, chronic inflammatory state, and embolic manifestations. Nevertheless, the precise nature of this relationship should still be clarified [7-9]. In addition, great difficulty can be experienced during the differential diagnosis of ghosts, as these findings tend to closely mimic vegetations and intracardiac thrombi on transesophageal echocardiography (TEE) [3,10,11].

Ghosts are predominantly reported in the setting of chronically implanted leads, whereas their occurrence after a very short period following device implantation and early explantation is rare and not well described in the literature, making the present case unusual and clinically relevant [6,7].

The differential diagnosis of these findings is crucially important, and multimodality imaging is required to accurately determine the true nature of residual masses in order to select appropriate management. TEE is considered the gold standard for detection, while cardiac magnetic resonance (CMR), positron emission tomography-computed tomography (PET-CT), intracardiac echocardiography (ICE), and computed tomography (CT) should also be considered [1,3,12]. The clinical importance lies in choosing the proper treatment, which might differ significantly, including follow-up control without treatment, prolonged antimicrobial administration, or surgical excision [3,7,13].

Here, we describe the case of a right-sided intracardiac “ghost” found after early ICD lead explantation due to methicillin-resistant Staphylococcus aureus (MRSA) bacteremia.

## Case presentation

A 55-year-old man with coronary artery disease and severe left ventricular systolic dysfunction (LVEF 35%) was under follow-up for ischemic cardiomyopathy. His medical history included hypertension, dyslipidemia, mild chronic kidney disease, and active smoking. An ICD had been implanted for the primary prevention of sudden cardiac death. Prior evaluation had demonstrated a chronic total occlusion of the left anterior descending artery with a large nonviable anterior wall scar on stress CMR, and he was managed conservatively with optimal medical therapy. Ten days after ICD implantation, he presented with malaise, low-grade fever, and mild erythema at the generator site. There was no discharge or swelling. He denied chest pain, dyspnea, or heart failure symptoms. On admission, he was febrile (38.2°C) but hemodynamically stable. Physical examination revealed no murmurs or peripheral stigmata of infective endocarditis. Laboratory investigations showed elevated inflammatory markers, with the full admission blood results summarized in Table 1. Multiple blood cultures grew MRSA, confirming bacteremia. Device-related infective endocarditis was suspected. The patient was started on intravenous antibiotics, and complete transvenous lead explantation was performed early during hospitalization under fluoroscopic guidance without complications.

**Table 1:** Laboratory values on the day of admission INR, international normalized ratio

Parameters	Value	Reference range
White cell count (×10⁹/L)	13.8	3.91-8.77
Neutrophils (×10⁹/L)	10.2	1.82-7.42
Lymphocytes (×10⁹/L)	2.88	0.85-3.00
Monocytes (×10⁹/L)	0.39	0.19-0.77
Eosinophils (×10⁹/L)	0.31	0.03-0.44
Basophils (×10⁹/L)	0.02	0.01-0.05
Platelets (×10⁹/L)	405	150-450
Hemoglobin (g/L)	12.1	11.9-15.4
Mean corpuscular volume (fL)	93	80-93.6
C-reactive protein (mg/L)	125	<5
Procalcitonin (ng/mL)	0.45	<0.5
Creatinine (mg/dL)	1.29	0.70-1.20
Urea (mg/dL)	37.4	16.6-48.5
Estimated glomerular filtration rate (mL/min/1.73 m²)	65	>90
Glucose (mg/dL)	125	74-109
Sodium (mmol/L)	133	136-145
Potassium (mmol/L)	4.2	3.4-4.5
Magnesium (mmol/L)	0.75	0.66-1.07
Calcium (mg/dL)	9.2	8.6-10.0
Albumin (g/dL)	3.8	3.5-5.2
Total protein (g/dL)	6.9	6.6-8.7
Lactic dehydrogenase (U/L)	209	135-214
Creatine phosphokinase (U/L)	202	<170
Creatine phosphokinase-MB (U/L)	19	<25
Bilirubin (mg/dL)	0.84	<1.20
Alanine aminotransferase (U/L)	35	10-50
Alkaline phosphatase (U/L)	58	40-129
Aspartate aminotransferase (U/L)	25	10-50
Gamma-glutamyl transferase (U/L)	33	10-71
N-terminal pro B-type natriuretic peptide (pg/L)	698	<125
Prothrombin time (s)	12.2	10.0-13.0
Activated partial thromboplastin time (s)	28.8	16.0-37.0
INR	0.99	0.85-1.15
D-Dimers (ng/m)	540	0.0-550.0

A follow-up TEE performed two days after ICD explantation revealed a large, highly mobile echodensity (30×15 mm) attached to the tricuspid valve, with an additional mass occupying part of the right atrial appendage, as demonstrated in Figures 1-3 and Video 1.

**Figure 1:**
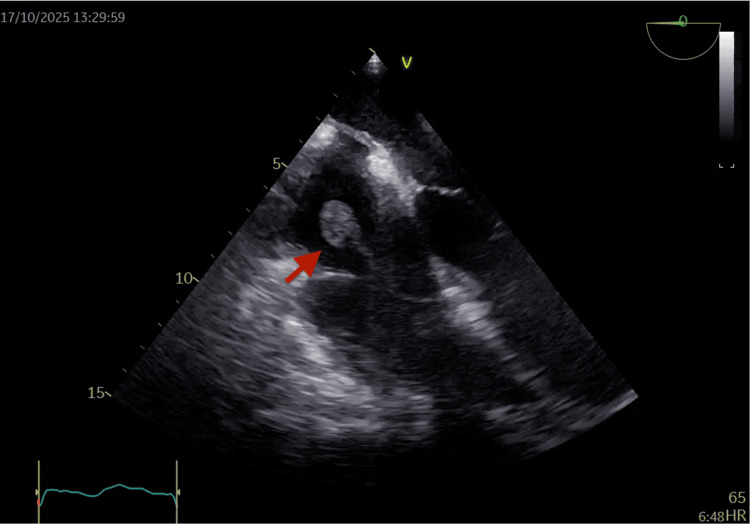
Mid-esophageal bicaval TEE image at 0° demonstrating a large, elongated, highly mobile echodense structure attached near the tricuspid annulus and extending into the right atrium TEE, transesophageal echocardiography

**Figure 2:**
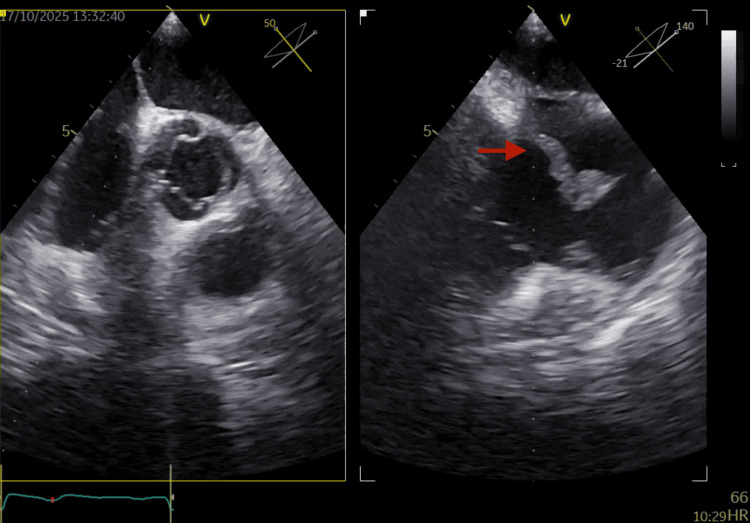
Mid-esophageal bicaval TEE image at 50° showing a tubular intracardiac structure within the right atrium TEE, transesophageal echocardiography

**Figure 3:**
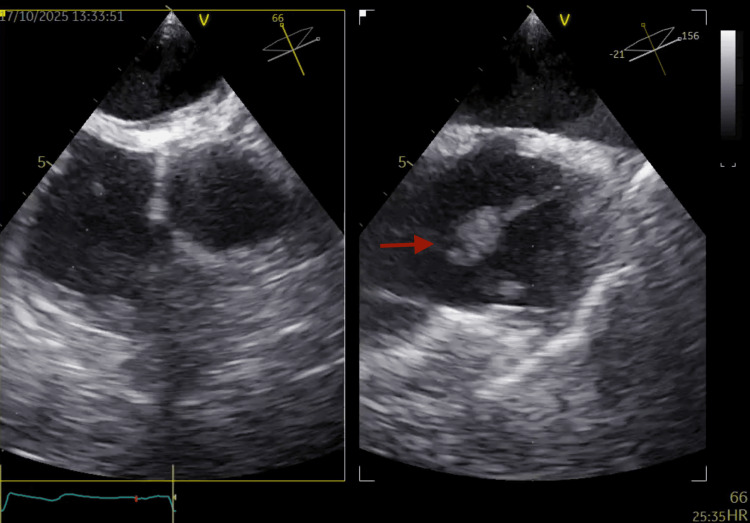
Transgastric right ventricular inflow TEE image at 66° confirming the filamentous, mobile configuration of the structure TEE, transesophageal echocardiography

**Video 1:** Mid-esophageal bicaval TEE video at 0° demonstrating a large, elongated, highly mobile echodense structure attached near the tricuspid annulus and extending into the right atrium TEE, transesophageal echocardiography

These findings raised the question of whether surgical management should be considered. A cardiothoracic surgical consultation was therefore sought, and further imaging was requested to better characterize the nature of these structures. CMR was performed one day after the initial TEE, including T1-weighted sequences and gadolinium-based contrast-enhanced imaging, which demonstrated multiple mobile intracardiac structures attached to the tricuspid valve, right atrial appendage, and superior vena cava, as shown in Figure 4 and Video 2.

**Figure 4:**
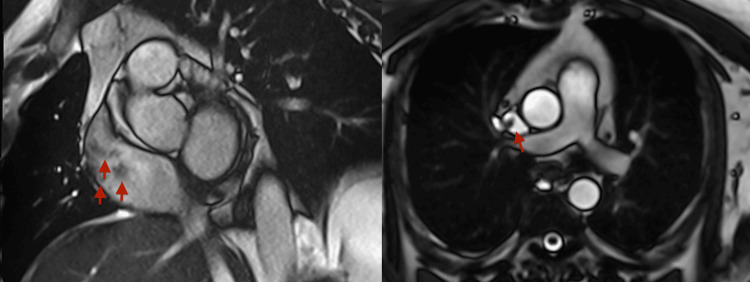
CMR images showing highly mobile formations within the right atrium, pedunculated and arising from the tricuspid valve, the right atrial appendage, and the superior vena cava. The structures demonstrate signal intensity similar to myocardium on T1-weighted sequences, without evidence of perfusion, and with heterogeneous contrast enhancement predominantly at their periphery CMR, cardiac magnetic resonance

**Video 2:** CMR video demonstrating highly mobile formations within the superior vena cava CMR, cardiac magnetic resonance

These structures demonstrated T1 signal intensity similar to myocardium, absence of first-pass perfusion, and peripheral late gadolinium enhancement, features consistent with an avascular organized fibrotic sheath rather than active infection. Overall, these findings supported the diagnosis of an intracardiac “ghost” secondary to fibrotic remnant formation following device extraction. In light of these findings and the patient’s clinical stability, surgical intervention was deferred. Computed tomography pulmonary angiography (CTPA) was also performed as a precautionary measure to evaluate for potential embolic phenomena arising from these structures. No evidence of pulmonary embolism was identified. Repeat echocardiography performed three days following the initial TEE demonstrated a marked reduction in the size of the mass, now appearing as a smaller tubular structure extending along the previous lead trajectory, as shown in Figure 5.

**Figure 5:**
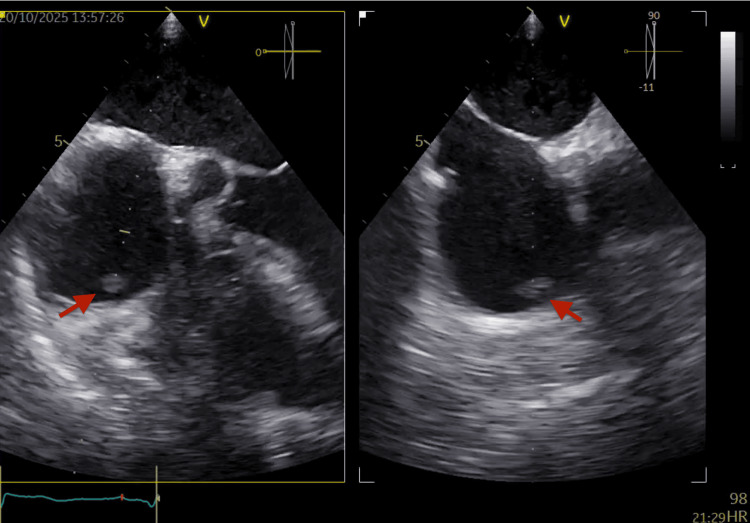
Mid-esophageal bicaval TEE image at 0° demonstrating a reduction in the size of the mobile echodense structure attached near the tricuspid annulus and extending into the right atrium TEE, transesophageal echocardiography

Given this interval regression, repeat CTPA was subsequently performed and revealed a new pulmonary embolism in the right lower lobe, along with a small pleural effusion. Anticoagulation therapy was initiated following this diagnosis, in keeping with standard management principles for provoked venous thromboembolism after recent device explantation. Although the pulmonary embolism may have been related to partial embolization of the intracardiac “ghost,” contributing to the observed reduction in mass size demonstrated in Figure 6, anticoagulation for three months was still considered appropriate given the confirmed provoked thromboembolic event and absence of contraindications.

**Figure 6:**
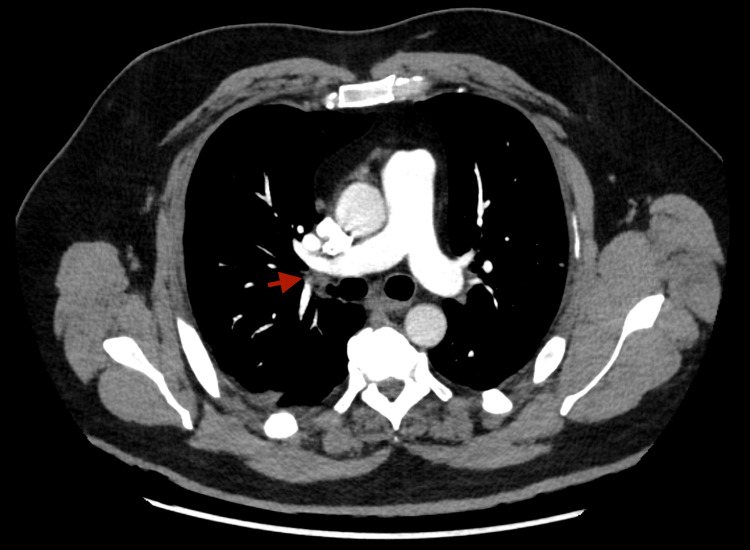
CTPA axial contrast-enhanced image demonstrating an intraluminal filling defect within a segmental branch of the right lower lobe pulmonary artery (arrow), suggestive of pulmonary embolism CTPA, computed tomography pulmonary angiography

Serial imaging over the following weeks demonstrated complete resolution of the previously observed intracardiac structures. The patient remained clinically stable, afebrile, and hemodynamically uncompromised, and surgical intervention was ultimately deemed unnecessary. He completed a six-week course of intravenous vancomycin, counted from the date on which blood cultures first became negative. At one-month follow-up, repeat TEE demonstrated no evidence of recurrent intracardiac structures, as shown in Figure 7. Blood cultures remained negative, inflammatory markers normalized, and the patient remained asymptomatic. He was discharged from the hospital with no further need for antibiotics. Further outpatient follow-up at two and three months confirmed sustained clinical stability with no recurrence of intracardiac masses or infectious features. Given the history of device-related infection, evaluation for a leadless defibrillator system was initiated as part of long-term postdischarge management to reduce the risk of recurrent device infection and lead-related complications.

**Figure 7:**
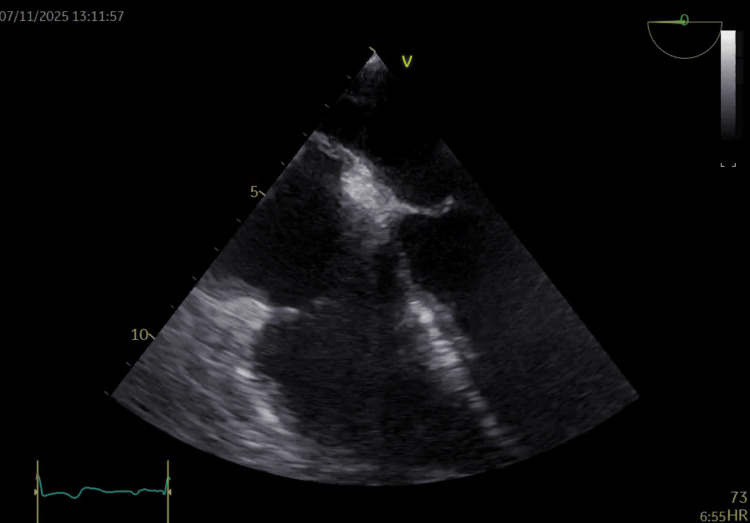
Mid-esophageal bicaval TEE image at 0° demonstrating the absence of the structure TEE, transesophageal echocardiography

## Discussion

"Ghosts" are mobile tubular intracardiac remnants that arise following TLE and tend to follow the path of the previous lead [3,5,6,14]. Despite becoming an increasingly common phenomenon recognized in contemporary electrophysiology practice, their precise composition, pathogenesis, significance, and management remain somewhat poorly understood [7,8]. Available literature suggests that ghosts represent remnant fibrous or fibrothrombotic sheaths that form as a consequence of interaction between the transvenous lead and the endocardium [5,6,13].

The mechanism of pathogenesis of ghosts is postulated to involve endothelial trauma induced by repetitive chronic movement of the lead, followed by activation of platelets, deposition of fibrin, thrombus organization, recruitment of inflammatory cells, and eventual fibrotic encapsulation of the structure with subsequent endothelialization [5-7,13]. As such, the sheath may gradually elongate and become tethered to intracardiac structures during lead extraction. The remnants of this sheath can remain attached to intracardiac structures even after hardware removal, resulting in preservation of the tubular shape of the lead, the so-called "ghosts" [3,5]. Ghosts have been mostly observed in the superior vena cava, right atrium, tricuspid valve, and right ventricle, which correspond to the areas where the lead is in contact with the endocardium [3,5,6]. Histopathologic analyses published in earlier literature have revealed various compositions of these structures, including collagen fibers, inflammatory infiltrates, thrombotic material, calcification, and fibrinous tissue residue, suggesting that their nature might change over time according to device longevity and level of inflammation [3,7,13]. A schematic illustration of the pathophysiology of ghost formation is shown in Figure 8.

**Figure 8:**
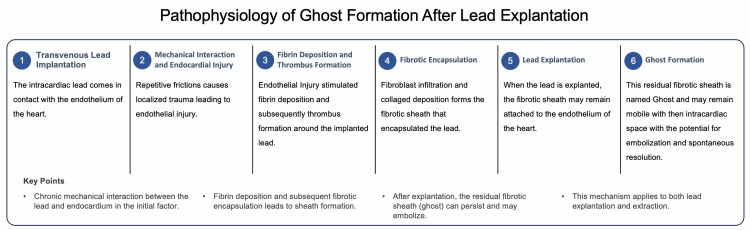
Schematic illustration of the pathophysiologic mechanism underlying “ghost” formation following transvenous lead explantation Chronic mechanical interaction between the intracardiac lead and endocardium promotes fibrin deposition and progressive fibrotic encapsulation. Following lead removal, a residual fibrotic sheath may persist, appearing as a mobile intracardiac structure (“ghost”), with the potential for embolization or spontaneous resolution. Image credit: Created by the authors using Microsoft PowerPoint (Microsoft, Redmond, WA).

The reported prevalence varies considerably according to imaging modality, timing of assessment, and patient population. Earlier retrospective studies using primarily postprocedural TEE reported incidences around 8%, whereas prospective studies with systematic multimodality imaging demonstrated rates closer to 14% [3,8]. More recent ICE-guided studies have reported even higher detection rates of up to 19%, and in selected cohorts undergoing systematic ICE evaluation immediately before and after extraction, ghost-like masses were identified in as many as 44-60% of patients, suggesting that smaller residual fibrotic sheaths may frequently remain underdiagnosed with conventional imaging alone [5,7-9]. Ghost formation appears more common in patients undergoing extraction for systemic infection or device-related endocarditis compared with noninfective indications [4,6-8,14]. On the contrary, the device type itself does not seem to significantly affect this phenomenon [6,14].

The clinical significance of ghosts is still under debate. Several research groups have shown that they might be correlated with higher long-term mortality after TLE, presumably due to persistent inflammation, endothelial dysfunction, or a prothrombotic state [8,9]. Nonetheless, there is no clear causality yet, and some researchers could not demonstrate any association with infections, recurrent cardiac complications, or mortality [4,7]. Accordingly, evaluation should be individualized based on the overall clinical picture.

From a diagnostic perspective, ghosts are one of the biggest challenges in echocardiography because of their close resemblance to vegetations, thrombi, or cardiac tumors [3,10,11]. TEE is the gold standard diagnostic imaging tool for evaluating the post-TLE state because of its superb spatial resolution and capability to assess lead and valve involvement [3,9]. ICE has also emerged as a valuable adjunctive imaging modality, allowing detailed real-time visualization of residual intracardiac sheaths and potentially improving detection of smaller ghost formations that may be underestimated by conventional echocardiography [5,7-9]. Nonetheless, echocardiographic features alone may not be sufficient for reliable differentiation of sterile fibrotic tissue from infective vegetation [3]. Thus, a multimodal imaging approach is often required in challenging cases.

In this particular case, CMR was crucial for making the final clinical decision. The structures showed T1 signal intensity typical of myocardium, absence of first-pass perfusion, and heterogeneous peripheral enhancement, features suggestive of organized fibrous rather than infective vegetations. Earlier literature stresses the importance of utilizing advanced imaging modalities for tissue characterization and ruling out an infectious process [3,12]. Furthermore, PET-CT and nuclear imaging techniques might prove valuable for identifying metabolically active processes around the residual masses or retained material [1,12].

The natural course of ghosts is highly unpredictable, with some remaining stable for many years and others resolving either gradually or acutely [15-17]. While the mechanisms responsible for regression of ghosts remain poorly understood, they may include endogenous fibrinolysis, structural remodeling, fragmentation, and/or embolization [18]. In our case, serial imaging performed later showed decreasing mass size followed by complete resolution. Notably, this evolution coincided with the detection of a pulmonary embolism, raising the possibility that partial embolization of the intracardiac structure may have contributed to its apparent regression. However, a causal relationship cannot be definitively established, and alternative causes such as infection-related embolization or coincidental venous thromboembolism (including deep vein thrombosis, prolonged immobility, recent device infection, central venous instrumentation, or a hypercoagulable state) are considered less likely, although they cannot be entirely excluded. Similar mechanisms have been hypothesized in the literature describing fragmentation or thrombotic dissolution of ghost-like structures [18]. Although many ghosts may follow a benign clinical course, careful monitoring and serial imaging follow-up are critical, particularly in patients with high-risk characteristics such as large, highly mobile masses, persistent inflammatory activity, residual infection, or evidence of embolization [2,5,12].

Management approaches must thus be tailored based on the clinical presentation, radiologic features, hemodynamics, microbiologic information, and risk of embolization [1,3,7]. Surgery can be considered in cases with persistence of infection, hemodynamic instability, large and mobile masses, recurrent episodes of embolization, or indeterminate diagnosis [11,17]. Nonetheless, in patients who are hemodynamically stable and have a noninfectious nature of the mass on multimodal imaging, nonsurgical management with imaging follow-up could be the preferred option. In this case, hemodynamic stability, absence of infection after follow-up blood cultures, and favorable imaging features demonstrating complete spontaneous resolution of the mass obviated the need for surgical intervention.

This case highlights the emerging importance of utilizing alternative devices after CIED infections. The use of leadless pacemakers or extravascular devices may reduce the risk of reinfection, particularly because they avoid intravascular components and eliminate the need for repeated transvenous access [1].

Learning points

Multimodality imaging is essential to distinguish postextraction “ghosts” from vegetations or thrombi, with CMR aiding tissue characterization and management. Not all cases require surgical intervention, and stable patients may be managed conservatively with close follow-up. Embolic complications, including pulmonary embolism, can occur during regression. Leadless or extravascular devices should be considered in patients with prior device-related infection.

Patient perspective

“I was worried when they said something was left behind after removing the device, but it was a relief to hear that it disappeared on its own. I’m glad the new device won’t have wires inside my heart.”

Figure 9 illustrates a summary of the patient’s journey.

**Figure 9:**
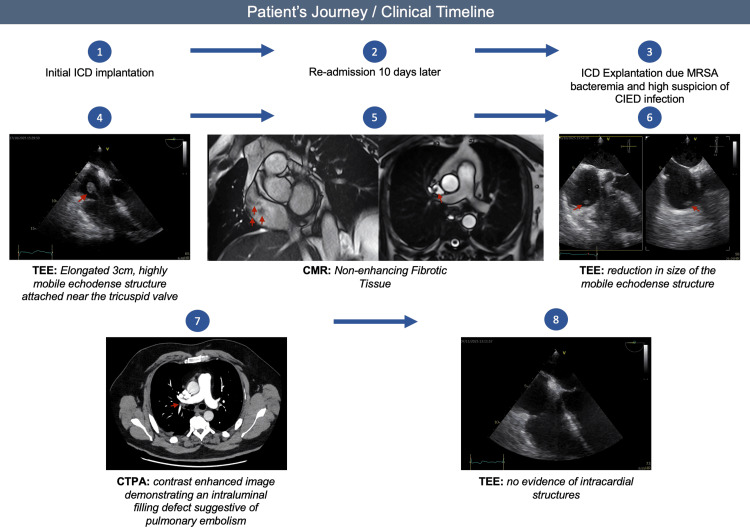
Patient’s journey/clinical timeline TEE, transesophageal echocardiography; MRSA, methicillin-resistant Staphylococcus aureus; CIED, cardiac implantable electronic device; ICD, implantable cardioverter-defibrillator; CMR, cardiac magnetic resonance; CTPA, computed tomography pulmonary angiography Image credit: Created by the authors using Microsoft PowerPoint (Microsoft, Redmond, WA).

## Conclusions

Intracardiac “ghosts” represent an uncommon but clinically relevant finding following TLE, often creating diagnostic uncertainty in the early postprocedural period. This case highlights the value of multimodality imaging, particularly CMR, in distinguishing fibrotic remnants from persistent infection and guiding management decisions. In selected clinically stable patients, a conservative approach may be appropriate, although the potential for embolic complications necessitates careful follow-up. Evolving device strategies, including leadless and extravascular systems, may further reduce the risk of recurrent infection in this population.
